# Overweight and obesity and associated factors among adult ART patients at Hawassa University Comprehensive Specialized Hospital, Southern Ethiopia

**DOI:** 10.1186/s40795-022-00556-1

**Published:** 2022-07-12

**Authors:** Ashenafi Kure, Amene Abebe, Daniel Baza, Wondimagegn Paulos

**Affiliations:** 1 Public Health Laboratory, Health Bureau, Southern Nations Nationalities and People’s Regional State, Hawassa, Ethiopia; 2 College of Medicine and Health Science, Department of Public Health, Wolaita Sodo University, Wolaita Sodo, Ethiopia; 3 Department of Pediatrics and Neonatal Nursing, Wolaita Sodo University, WolaitaSodo University, Wolaita Sodo, Ethiopia; 4 College of Medicine and Health Science, Department of Human Nutrition, Wolaita Sodo University, Wolaita Sodo, Ethiopia

**Keywords:** Anti-Retroviral Therapy, BMI, HIV/AIDS, Obesity, Overweight, Patient

## Abstract

**Background:**

Overweight and obesity complicates the care and treatment of ART patients and predispose them to chronic non-communicable diseases. However, there is a shortage of research evidence on overweight and obesity and its associated factors among adult ART patients in our setting. Therefore, this study aimed to asses overweight and obesity and associated factors among adult ART patients at Hawassa University Comprehensive Specialized Hospital, Southern Ethiopia.

**Methods:**

A facility-based cross-sectional study design was conducted by using systematic sampling technique. Primary and secondary data were collected from 369 adult ART patients from February to May 2017. Structured interviewer-administered questionnaire and laboratory outputs were used as primary data. The patient’s baseline medical records were used as secondary data. Ethiopian Ministry of Health ART patient’s follow-up tool was used to collect the required information. The standard laboratory and well-calibrated digital Seca Scale and portable Stadio-meter were used to collect medical and anthropometric data. Data were entered into Epi- data version 3.1 and exported to SPSS version 20 for analysis. Descriptive statistics were calculated and presented by tables, graphs and texts. Binary and multivariable logistic regression analyses were computed and the level of statistical significance was declared at *p*-value < 0.05.

**Results:**

The prevalence of overweight and obesity (BMI ≥ 25 kg/m2) was 43.4% (95% CI = 43.35, 43.45). The difference in the overweight and obesity between the study period and initial commencement of ART was 35%. The course of HIV chronic care since the commencement of ART and during the study was 35%. Higher recent CD4 counts (200-499cells/mm3) (AOR = 3.15, 95%CI = 1.04–9.49) and (≥ 500 cells/mm3) (AOR = 7.58, 95%CI = 2.49–23.08), hypertension (AOR = 2.57, 95%CI = 1.24–5.35), higher baseline BMI status (AOR = 5.93, 95%CI = 2.62–13.40) and abdominal obesity (AOR = 1.82, 95%CI = 1.07–3.10) were significantly associated with overweight and obesity.

**Conclusion:**

In this study, a high prevalence of overweight and obesity among adult ART patients was reported compared to general adult population in Ethiopia. Overweight and obesity were significantly higher among hypertensive, with higher recent CD4 counts and abdominal obese ART patients. Thus, screening of overweight and obesity, incorporating nutritionist/dietician into the routine chronic care, and regular monitoring of the nutritional status of ART patients is recommended.

**Supplementary Information:**

The online version contains supplementary material available at 10.1186/s40795-022-00556-1.

## Background

Overweight and obesity are the results of an energy imbalance. Bodyweight tends to remain the same when the number of calories eaten equals the number of calories the body uses or “burns”. Over time, when people eat and drink more calories than they burn, the energy balance tips towards weight gain, overweight, and obesity [1]. Overweight and obesity are risk factors for type 2 diabetes, cardiovascular diseases, high blood pressure, non-alcoholic fatty liver diseases, osteoarthritis, some types of cancer, kidney disease, stroke, social, and psychological impacts [2–5].

Currently, there is no cure for HIV/AIDS but its treatment typically involves Anti-Retroviral Therapy (ART) [6, 7]. After the introduction of ART, AIDS patients' care and treatment have significantly improved. ART reduces morbidity and mortality, creates enabling conditions for more effective control of the new HIV infections, improves restoration of immunity, and increased the life expectancy; turning AIDS into a chronic disease [7–9]. However, overweight and obesity and its associated health problems among ART patients become the unfinished public health challenge [9].

In 2015, there were 36.7 million people living with HIV worldwide, of which the Sub-Saharan Africa (SSA) region remains the worst affected. The global coverage of ART reached 46% at the end of 2015 [10, 11]. According to HIV-related estimates and projections for Ethiopia, the national prevalence of HIV infection in 2016 was 1.1% among the adult population [12].

There is high prevalence of overweight and obesity among ART patients in both developed and developing countries [13–18]. Prolonged treatment duration, advanced stages of the AIDS disease, and the use of certain drug classes such as protease inhibitors (PI) are identified to be the main risk factors for the development of metabolic abnormalities among ART patients [19].

The rapid progression of pre-ART underweight or normal weight adult ART patients to overweight and obesity after the commencement of the therapy and increasing rates of overweight and obesity is a potential concern in adult ART patients. Although, women and men are equally overweight, women are more obese than men during the therapy [13–15].

Overweight and obesity which previously considered as the main health problem of high-income countries is now a days is an increasingly important public health concern in Sub-Saharan Africa (SSA) region [20]. In Ethiopia, like any other developing countries, overweight and obesity were previously associated with diabetes and high income earning, but since recently, these conditions are becoming more common in the general population, aggravating the poles of the burden of poverty and hunger in one aspect and overweight and obesity on the other hand [21–24].

Overweight and obesity is a predisposing factor for several underlying chronic non-communicable diseases which complicates the care and treatment of ART patients. Though, overweight and obesity among ART patients is an emerging public health concern, it is left unknown fully [8, 14, 15]. Moreover, in Ethiopia in general and the study setting specifically, comprehensive data on the magnitude and progression of overweight and obesity and associated factors among adult HIV patients on ART is scarce. Therefore, the aim of this study was to assess overweight and obesity and associated factors among adult ART patients in Hawassa University Comprehensive Specialized Hospital, Southern Ethiopia.

## Methods and materials

### Study design, setting and study period

A facility-based cross-sectional study design was employed. In this study, cross-sectional study design was applied because the study intended to assess the cause and effect at the same point in time. Hawassa University Comprehensive Specialized Hospital HUCSH is a teaching referral Hospital of Hawassa University, Ethiopia. HUCSH is found in Hawassa city, capital of Southern Nations and Nationalities and People’s Regional State (SNNPRS) and it is located about 275 km from Addis Ababa, the capital city of Ethiopia in the Southern direction. It is one of the main Hospitals providing chronic HIV care and ART services in the southern region, Ethiopia. According to the information obtained from the Hospital, at the time of the study, there were 7,126 ever enrolled HIV-positive adult patients in HIV chronic care in the Hospital, out of which 2,518 were receiving ART. The study was conducted from February to May 2017.

### Sample size determination and sampling procedures

The sample size for the study was calculated using single population proportion formula. The prevalence of overweight and obesity 32.1% from a similar study conducted in Rio de Janeiro, Brazil among HIV infected patients [25], 95% confidence level of significance (Zα/2 = 1.96) and margin of error 5% are used calculate the sample size (335). To make sure sample size sufficiency, using Open epi Info version 3.03, the sample size was calculated using significantly associated covariates from other related studies. Thus, using age (older age) (137) [16], sex (female sex) (135) [16], and being diabetic (209) [25]. However, the total sample size calculated using the prevalence of overweight and obesity (335) was higher than the sample size calculated using the covariates. So that, the final sample size was taken as 335. After adding 10% (34 participants) for non-response, it became 369.

A systematic sampling technique was used to select the 369 study participants. According to the two weeks (January 4–18, 2017) Hospital ART register report, on average, 35 (25–45) patients have visited the ART clinic daily. Considering 3 months of the study period (22 working days per month) starting from February 15, 2017, about 2,310 patients were estimated to visit the Hospital. Using the sample size 369, the sampling interval was estimated to be 6. Of the first 6 participants who visited the Hospital on February 15, 2017, one patient was randomly selected by lottery method; then every 6^th^ patient was included in the study until May 15, 2017. Only study participants whose age 18-year-old or more with complete weight and height records at the commencement of the study were included. The source population for the study were all people living with HIV (PLWH) and in HIV chronic care and treatment in HUCSH. ART patients with age 18-year-old and above and with complete weight and height records at the commencement of the study were included in the study. Study participants with missing initial CD4 (Cluster of Differentiation four positive T-lymphocyte cells) count data, pregnant at the time of data collection, seriously ill, and/or with spinal deformity were excluded from the study.

### Data collection tools and procedures

Ethiopian, Ministry of Health HIV care/ART follow-up tool was used to collect the required data [26]. Structured interviewer-administered questionnaire and laboratory outputs collected during data collection were used as primary data. The patient’s baseline medical records were used as a secondary data. The data extraction/collection tool included socio-demographic and economic data (age, sex, marital status, ethnicity, educational level, residence, monthly income, and occupational status); medication and health status data (functional status, duration on ART in months, drug adherence status, length of time since HIV diagnosis confirmed, type of ART, WHO (World Health Organization) clinical stage, CD4 counts in cells/mm3, hypertension in mm Hg, hemoglobin in mg/dl, fasting blood glucose in mg/dl, history of TB and history of opportunistic infections and abdominal obesity) and anthropometric data (weight (kg), height (m), and BMI (Body Mass Index) (kg/m2) were recorded from the patients’ medical record and measured based on the status of the patients at the initiation of ART and the start of the study accordingly [5, 26] (Additional file 1).

Anthropometric, medication, and health and laboratory data were gathered at two instants; baseline data from patient’s follow-up registry and during the study. Height was measured using a Stadio-meter and recorded to the nearest 0.5 cm. Weight was measured to the nearest 0.1 kg using a well-calibrated, portable digital scale (Seca electronic scale, 770 Hamburg). Both waist and hip circumferences were measured using the SECA ® (SECA, Germany) non-stretchable tape to the nearest 0.1 cm. The digital sphygmomanometer apparatus was calibrated and standardized to measure blood pressure. Two blood pressure measurements were measured and the average was taken. The first measurement was made after 10 min of rest by the participant after arrival and the second measurement was made after a one-minute interval of the first measurement. Both measurements were made in a sitting position and the right upper arm was placed at the level of the heart [27].

Current fasting blood glucose, hemoglobin level, and CD4 counts were measured from 6 ml fasting blood samples drawn from the vein by a trained medical laboratory technologist. Fasting blood glucose was measured using Prodigy Auto Code® Talking blood glucose monitoring system (Prodigy Diabetes Care, LLC 2701-A Hutchison McDonald, Charlotte, North Carolina) and Current CD4 counts were measured by using a flow-cytometry instrument (Becton–Dickinson, CA, USA) and categorized according to its clinical significance [28]. Hemoglobin was measured with a Cell Dyne hematology analyzer (US).

### Data quality control and standardization

Questionnaire was first prepared in English and translated to Amharic language by language experts then back translated to English to maintain its consistency. Both data collectors and supervisors were trained by principal investigator for three days on the objective, relevance of the study, the operation of the weight and height measurement scales, laboratory investigation procedures and interviewing approach, criteria’s and procedures of participants selection, respondent’s right, proper filling of the questionnaire and data recording. The questionnaire was pre-tested on 5% of the sample size in Adare Hospital, Hawassa, Ethiopia. Based on the pre-test, validity and reliability of the measurement was checked, questions that posed difficulty were revised and modified, but those found to be unclear or confusing were removed.

To assure the reliability of anthropometric measurements, standardization test was done on five ART patients prior to actual data collection. To perform the standardization, first the five ART patient’s weight and height was measured by the experts. Then, the anthropometric data collectors measured the same ART patients twice with some time intervals. The anthropometric data then entered in to ENA SMART software to see relative Technical Error of Measurements (TEM). The TEM output was compared with the acceptable range for relative TEM using beginner anthropometric value for inter-examiners and was found to be in acceptable range, < 2.0%.

Anthropometric parameters (height and weight) were measured by trained data collectors with the participants of the study wearing light clothing and no shoes; standing erect, and looking straight in a vertical plain. Prior to measuring weight, the scale was checked for zero reading and standardization was done according to WHO recommendations placing standard calibration weight of 2 kg iron bar on the scale to ascertain accuracy. If the scale weight did not match with calibration weight, the scale was calibrated by adjusting its calibration screw, while the calibration weight was on the scale. Both weight and height measurements were taken twice and the average was recorded. Waist circumferences were obtained by measuring the distance around the smallest area below the rib cage and above umbilicus (belly button). Hip circumference measurements were taken at the point yielding the maximum circumference over the buttocks with the tape in a horizontal plane, touching but not compressing the skin. Both waist and hip circumference measurements were carried out twice and the average of the two readings was recorded as the final measurement to calculate the waist-to-hip circumference.

Furthermore, data collection process was strictly followed on daily basis by the supervisor and the principal investigator. Completeness, accuracy and consistency of the collected data were checked on daily bases during data collection by the supervisors and principal investigator. Standard operating procedures were adhered during sample collection, processing, transportation, and detection of Anaemia, Hypertension, DM, and CD4 counts.

### Data processing and analysis

Data were coded and entered into Epi-Data version 3.1 statistical software and the data were exported to SPSS (Statistical Packages for Social Science) for windows version 20 (IBM SPSS Statistics, IBM Corp, New York) for analyses. Descriptive statistics using frequency with proportions mean and standard deviations were used to present summary statistics. All continuous variables were checked for normality by using the Kolmogorov–Smirnov test at *p*-value > 0.05. Before inclusion of predictor variables, multicollinearity was also checked among selected variables at a cut-off point of VIF < 10 and tolerance test greater than 0.1.

Bivariate and multivariate logistic regression was done to assess the association between overweight and obesity and demographic, medication and health status, and anthropometric variables. Hosmer and Lemeshow goodness of fit test was used to assess the fitness of the model during multivariate analysis at *p*-value > 0.05. All variables which were significant in the bivariate analyses at *p*-value < 0.25 were included in the multivariate logistic regression model and their independent and significant association has been assessed while controlling for the possible confounding variables. Strength of association was measured using both crude and adjusted odds ratios along with a 95% confidence interval. *P*-value < 0.05 was considered as a cut-off point to declare a statistically significant association of dependent variables with the independent variables.

### Operational definition of terms and measurements

Clinical stages (I–IV): defined based on the WHO classification for AIDS patients [29].

Overweight and obesity: defined based on WHO classification; BMI 25–29 kg/m^2^ overweight and BMI > 30 kg/m^2^ obese [4].

Hypertension: defined as systolic/diastolic blood pressure >  = 140 mmHg /90 mmHg [27].

History of hypertension: is previously diagnosed hypertension and taken from the medical record of the patients.

Abdominal obesity: defined Based on the WHO classification of Waist-Hip-Ratio (WHR) in men (> 0.9 or waist circumference >  = 102 cm) and in females (> 0.85 or waist circumference >  = 88 cm) [5].

Anaemia: Haemoglobin concentration of below 13 g/dl in males and below 12 g/dl in females respectively were graded as anaemic [30].

## Results

### Socio-demographic and economic characteristics of the study participants

A total of 369 adult ART patients were participated in the study making a response rate of 100%. The majority 229 (62.1%) the study participants were females and the rest were males. The mean age of the study participants was 35.9 with a standard deviation of 9.2 and 277 (75.1%) of the participants were in the age range of (18 -39 years). A very high proportion 361 (97.8%) of the study participants were urban residents and slightly higher than half 186 (50.4%) of the study participants were married. Regarding educational status, 125 (33.9%) and 118 (32.0%) were completed secondary and primary education respectively. Concerning their occupations, 338 (91.6%) of the study participants were employed. Regarding religion, 220 (59.8%) were Orthodox Christianity religion followers. Over half 198 (53.7%) of the study participants earn less than one thousand Ethiopian Birr (< 1000 ETB) per month. At the commencement of ART only 31 (8.4%) patients were overweight and obese (Table 1).

**Table 1:** Socio-demographic and economic characteristics by overweight and obesity among adult ART patients in Southern Ethiopia, 2017

			**Overweight and Obese**			
	**All patients**		**No**		**Yes**	
	**Number**	**%**	**Number**	**%**	**Number**	**%**
**All patients**	369	100	209	56.6	160	43.4
**Age (Years)**						
18–39	277	75.1	161	58.1	116	41.9
≥ 40	92	24.9	48	52.2	44	47.8
**Sex**						
Male	140	37.9	74	52.9	66	47.1
Female	229	62.1	135	59	94	41.0
**Marital status**						
Married	186	50.4	107	57.5	79	42.5
Unmarried	183	49.6	102	55.7	81	44.3
**Educational status**						
No formal education	48	13	29	60.4	19	39.6
Primary School	118	32	73	61.9	45	38.1
Secondary School	125	33.9	68	54.4	57	45.6
Tertiary School	78	21.1	39	50	39	50
**Employed**						
No	31	8.4	17	54.8	14	45.2
Yes	338	91.6	192	56.8	146	43.2
**Monthly income (ETB)**						
< 1000	198	53.7	124	62.6	74	37.4
≥ 1000	171	46.3	85	49.7	86	50.3
**Place of residence**						
Urban	361	97.8	203	56.2	158	43.8
Rural	8	2.2	6	75	2	25
**Religion**						
Orthodox Christians	220	59.8	125	56.8	95	43.2
Muslim	30	8.2	19	63.3	11	36.7
Protestant Christians	109	29.6	61	56	48	44
Other^a^	9	2.4	4	44.4	5	55.6
**Ethnicity**						
Sidama	40	10.8	21	52.5	19	47.5
Wolaita	61	16.5	37	60.7	24	39.3
Oromo	69	18.7	35	50.7	34	49.3
Guragie	39	10.6	23	59	16	41
Other^b^	160	43.4	93	58.1	67	41.9
**Baseline BMI of patients**	369	100	338	91.6	31	8.4

^a^Catholic, Adventist, Apostolic ^b^kembata, Hadya, Silte, Dawuro, Gedeo, Amhara, Tigre, Gamo, Konso, Halaba

### Prevalence of overweight and obesity and its progression in the course of ART among adult ART Patients

**Fig. 1:**
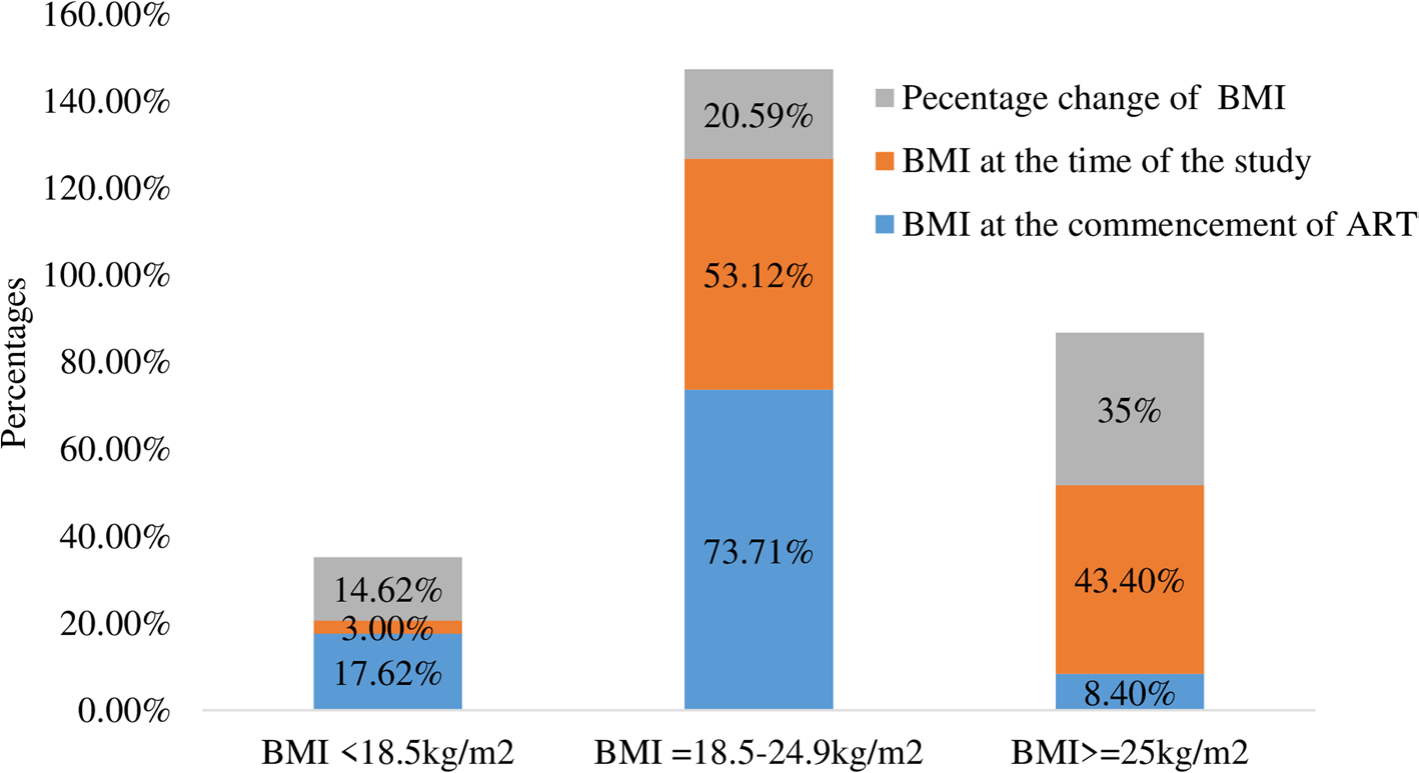
Changes in BMI among adult ART patients in Southern Ethiopia, 2017

The overall prevalence of overweight and obesity (BMI ≥ 25 kg/m2) in this study was 43.4% (95% CI = 43.35**, **43.45). A high proportion of overweight and obesity was reported among hypertensive study participants 36 (65.5%) followed by participants whose recent CD4 counts ≥ 500 cells/mm399 (49.5%). Overweight and obesity among abdominal obese study participants was 116 (48.1%). The prevalence of overweight and obesity and underweight at the commencement of ART were 8.4% and 17.6% respectively. At the commencement of ART a higher proportion of overweight and obesity was reported among women (4.6%) than men (3.8%). Regarding underweight, higher reduction 17.6% to 3% and higher progression in the overweight and obesity 8.4% to 43.3% was reported in the current study. Overweight and obesity among women (26.6%) during the study was higher when compared to the proportion (18.6%) among men participants. The average duration of the participants on ART at the time of the study was 7.1 years with a standard deviation of 5.9 years. In the overall course of care since the commencement of ART 188 (50.9%) of the participants gained weight, 169 (45.8%) no change in their weight, and 12 (3.3%) lost their weight (Fig. 1).

### Factors associated with overweight and obesity among adult ART patients

Bivariate and multivariable logistic regression analyses was computed; in the bivariate analysis monthly income, recent CD4 counts, fasting blood glucose, history of hypertension, blood pressure (hypertension), anaemia, BMI at the commencement of ART and abdominal obesity (WHR) were found to be significantly associated at (*p*-value < 0.05).However, in the multivariable logistic regression analyses, controlling for the possible confounders, CD4 counts at the commencement of ART (200-499cells/mm3), recent CD4 counts (200-499cells/mm^3^), systolic to diastolic blood pressure at 140 mm Hg/90 mm Hg, baseline BMI status and abdominal obesity were found to be statistically significantly associated with overweight and obesity.

ART patients whose CD4 counts 200-499cells/mm3 at the commencement of ART were 44.6% less likely to be overweight and obese when compared to those whose CD4 counts at the commencement was < 200 cells/mm^3^ (AOR, = 0.554, 95% CI = 0.327–0.939) and ART patients whose CD4 counts were 200-499cells/mm^3^ at the time of the study were more than 3 times more likely to be overweight and obese than those whose CD4 counts were < 200 cells/mm^3^(AOR = 3.15,95% CI = 1.04–9.49)). ART patients whose CD4 counts ≥ 500 cells/mm^3^ at the time of the study were more than 7 times more likely to be overweight and obese than whose CD4 counts were < 200 cells/mm^3^ (AOR = 7.58, 95% CI = 2.49–23.08) (Table 2).

**Table 2:** Overweight and obesity and associated factors among adult ART patients in Southern Ethiopia, 2017

Variables	Overweight and obese		COR (95%CI)	AOR (95%CI)
	No	Yes		
Educational status				
No formal education	29	19	1	1
Primary School	73	45	0.941 (0.473–1.871)	1.012 (0.452–2.267)
Secondary School	68	57	1.279 (0.650–2.518)	1.450 (0.651–3.229)
Tertiary School	39	39	1.526 (0.736–3.165)	1.072 (0.438–2.623)
Monthly income				
< 1000(ETB)	124	74	1	1
≥ 1000(ETB)	85	85	1.695 (1.119–2.569) *	1.297 (0.749–2.246)
CD4 counts at ART commencement				
< 200 cells/mm^3^	122	104	1	1
200-499cells/mm^3^	78	52	0.782 (0.505–1.211)	0.554 (0.327–0.939) *
≥ 500 cells/mm^3^	9	4	0.521 (0.156–1.742)	0.341 (0.083–1.402)
Recent CD4 Count				
< 200 cells/mm^3^	26	7	1	1
200–499 cells/mm^3^	82	54	2.446 (0.992–6.031)	3.148 (1.044–9.491) *
≥ 500 cells/mm^3^	101	99	3.641 (1.511–8.772) *	7.583 (2.492–23.081) *
History of DM				
No	207	154	1	1
Yes	1	6	8.104 (0.966–68.005)	5.434 (0.398–74.165)
Fasting Blood Glucose				
Not diabetic	175	118	1	1
Diabetic	34	42	1.832 (1.101–3.047) *	1.309 (0.691–2.480)
History of hypertension				
No	207	150	1	1
Yes	2	10	0.145 (0.031–0.671) *	0.328 (0.059–1.839)
Blood pressure ≥ 140 mm Hg or 90 mm Hg)				
No hypertension	190	124	1	1
Hypertensive	19	36	2.903 (1.593–5.290) *	2.575 (1.238–5.355) *
Anaemia				
No anaemia	162	142	1	1
Anaemic	47	18	0.437 (0.243–0.787) *	0.569 (0.287–1.129)
Stavudine (D4T) use				
No	158	130	1	1
Yes	51	30	1.399 (0.842–2.323)	2.907 (1.528–5.533)
Drug adherence				
Good	194	156	2.010 (0.385–10.502)	0.422 (0.029–6.108)
Fair	10	2	0.500(0.054–4.672)	1.282 (0.187–8.802)
Poor	5	2	1	1
Baseline BMI Status				
< 18.5 kg/m^2^	56	9	1	1
18.5–24.9 kg/m^2^	145	128	5.493 (2.613–11.546) *	5.930 (2.625–13.396) *
25–29.9 kg/m^2^	8	23	17.889 (6.143–52.098) *	19.908 (6.114–64.817) *
Abdominal obesity (WHR)				
No	84	44	1	1
Yes	125	116	1.772 (1.137–2.761) *	1.818 (1.067–3.096) *

^*^ *P*-value < 0.05

This study also revealed that ART patients whose blood pressure (systolic and/or diastolic blood pressure ≥ 140 mm Hg/90 mm Hg) were approximately 3 times more likely to be overweight and obese than those whose blood pressure (systolic and/or diastolic blood pressure < 140 mm Hg/90 mm Hg) (AOR = 2.57, 95% CI = 1.24–5.35). BMI status at commencement ART was also a significant predictor of overweight and obesity. Being overweight and obese was approximately 6 times higher among ART patients whose BMI at the time of commencement of ART was in normal weight range, when compared to ART patients whose BMI at the commencement of ART was < 18.5 kg/m^2^ (underweight) (AOR = 5.93, 95%CI = 2.62–13.40). The current study also shows that the odds of being overweight and obese was statistically approximately 2 times significantly higher (AOR = 1.82, 95%CI = 1.07–3.10) among ART patients of abdominal obesity (Table 2).

## Discussion

The overall prevalence of overweight and obesity among the study participants in this study was found to be 43.4% (95% CI = 43.35, 43.45). The prevalence in this study is far higher than the general population in Ethiopia, 20.4% [20, 31] and the study result of Rio de Janerio, Brazil, 32.1% [25]. This might be owing to HIV treatment complications and drug side effects in ART patients. This prevalence is in line with the study report of University of Alabama at Birmingham where the study reports the prevalence of 44% [32]. The prevalence in this study is lower than the study report of Rio de Janeiro, Brazil [33],.S. Naval Medical Center in San Diego, USA [14] and in England [34] in which 50%, 63% and 67% of prevalence overweight and obesity had been reported. The observed differences in the prevalence might be explained by the difference in the sample size and socio-demographic and economic variables.

In the current study, the percentage change of overweight and obesity over the course of HIV chronic care since the commencement of ART increased from 8.4% to 43.4%. The difference is much more higher than the study results of Abidjan, Côte d’Ivoire, 27.6% to 35% [35] and in USA Naval center 54% to 63% [13]. The possible explanation for this higher discrepancy might be due to socio-economic status, healthcare delivery system and socio-cultural variations.

In this study, weight gain was observed in 50.9% of ART patients over the course of HIV chronic care. This result is lower than the study report of USA, 62%. [13]. The possible reason for the difference might be due to socio-economic, dietary habit and healthcare delivery system differences. Certain evidence also reveal that the weight gain among PLWH might be an indication of the efficacy of ART and/or higher calorie intake owing to patients desire not to appear too thin which could lead others suspect their HIV status [14, 15, 36].

In the current study, the prevalence of abdominal obesity (WHR) among ART patients was (65.3%). This result is higher than the result observed in Malaysia, 36.5% [37]. The possible reason for the difference might be the time lapse between the two studies. But, both studies reported that higher prevalence of abdominal obesity in the respective study setting and time. The high prevalence of abdominal obesity among ART patients might be due to the fact that morphological alterations including reduction in hip circumference along with an increase in waist circumference owing to the side-effects of ART medications [33, 38].

ART patients whose CD4 counts 200-499cells/mm^3^ were more than 3 times more likely to be overweight and obese than those whose CD4 counts were < 200 cells/mm^3^ (AOR = 3.15, 95% CI = 1.04–9.49)) and ART patients whose CD4 counts ≥ 500 cells/mm^3^ were more than 7 times more likely to be overweight and obese than those whose CD4 counts < 200 cells/mm^3^ (AOR = 7.58, 95% CI = 2.49–23.08). Similar result had been reported in a study conducted in Tanzania [16]. This result might be justified by the fact that immune restoration and viral suppression after ART might have more contribution for effective control of AIDS related morbidity and increased chance of being over and obese [33].

This study also demonstrated that hypertensive patients are approximately three times higher risk of being overweight and obese when compared to their counter parts (AOR = 2.57, 95% CI = 1.24–5.35). This result is corroborated with studies done in South Africa and USA [39, 40]. The possible explanation for this finding might be related to the combined effect of anti-hypertensive and ART drugs [41]. In this study, ART patients with abdominal obesity are around 2 times more likely to be overweight and obese than their counter parts (AOR = 1.818; 95%CI = 1.067–3.096) This result was reported similarly with studies in USA where it reports relationship between BMI and abdominal fat accumulation is linear (*p* < 0.001) [42, 43].

The findings of this study also revealed that ART patients who were overweight and obese at the commencement of ART were 19 times and normal weight 5 times respectively were more likely to be overweight and obese when compared to underweight. This result confirms the results of the studies conducted in Dare Salaam, Tanzania and Malaysia [16, 37]. This might be because of ART patients who were overweight and obese or normal weight at the commencement of ART are closer for further obesity than their counter parts.

According to this study findings, policy makers, governmental and non-governmental organizations and other stakeholders who are working on HIV/AIDS prevention and control should emphasize on the follow–up of nutritional status and dietary habits of ART patients. Prevention and control of hypertension and follow-up of patients who are overweight and obese during the commencement of ART is recommended. Healthcare workers and other stakeholders should focus on providing continuous health education regarding controlling body weight by healthy eating habits and regular physical exercise as patients CD4 count increases. In addition, screening of overweight and obesity, incorporating nutritionist/dietician in to ART patients chronic care team and monitoring of nutritional status of the patients are recommended. Furthermore, we recommend further studies using mixed methods including household wealth index of participants and nutritional factors such as dietary diversity and food habits.

## Conclusions

In this study, higher prevalence of overweight and obesity at the time of the study and higher difference of the prevalence at the time of the study and at the commencement of the patients to ART was reported. The prevalence and the difference of the prevalence are a major public health concern when compared to various studies in sub-Saharan Africa region. Overweight and obesity were significantly higher among hypertensive, with higher recent CD4 counts, higher BMI at the commencement of ART and abdominal obese ART patients.

### Study strengths

This study has covered the prevalence of overweight and obesity and associated factors among adult ART patients which was not well addressed previously. Moreover, in this study, important medical and anthropometric measurements were determined by using standard procedures and tools.

### Study limitations

This study has limitations. First, as the study sample consisted of adult ART patients who came to the hospital and therefore we cannot generalize our findings to other districts elsewhere in Ethiopia or other sub-Saharan developing countries. Second, nutritional and household wealth index factors were not assessed which might have an association with overweight and obesity of the patients. Third, data was collected on exposures and outcomes simultaneously, thus association of variables identified but it cannot establish causal relationships.

## Supplementary Information

**Additional file 1. **Participant information sheet, consent form and tool English version.

## Data Availability

The datasets during and/or analysed during the current study are available from the corresponding author upon reasonable request.

## Abbreviations

AOR: Adjusted Odds Ratio
ART: Antiretroviral Therapy
AIDS: Acquired Immune Deficiency Syndrome
BMI: Body Mass Index
CD4: Cluster of Differentiation four positive T-lymphocyte cell
CI: Confidence Interval
ETB: Ethiopian Birr
HUCSH: Hawassa University Comprehensive Specialized Hospital
HIV: Human Immunodeficiency Virus
OR: Odd Ratio
PLWH: People Living With HIV
SPSS: Statistical Package for Social Science
WHO: World Health Organization
