# Supplementary material for: Overweight and obesity and associated factors among adult ART patients at Hawassa University Comprehensive Specialized Hospital, Southern Ethiopia
Source: BMC Nutr. 2022 Jul 12;8:62. doi: 10.1186/s40795-022-00556-1 (PMC9275139; doi:10.1186/s40795-022-00556-1)
Supplement: Supplementary file 1 — Additional file 1. Participant information sheet, consent form and tool English version. [file 40795_2022_556_MOESM1_ESM.docx]

## Participant information sheet, consent form and tool English version

**Title:** **Overweight and Obesity and Associated Factors among Adult ART Patients at Hawassa University Comprehensive Specialized Hospital, Southern Ethiopia**

**Introduction:**

Hello my name is -------------I am data collector for a study conducted by researchers from Wolaita Sodo University college of Health Science and Medicine and School of Public Health**.** This information sheet is prepared by group of researchers whose main aim is to determine the prevalence and associated factors of overweight/obesity among adults receiving ART in HUCSP in ,Southern Ethiopiafrom February – May; 2017.

This study will be done by a research group which includes one principal investigator, laboratory professionals from HUCSH laboratory , ART Nurses and advisors from Wolaita Sodo University , School of Public Health. In this study prevalence and associated factors of overweight and obesity among adults receiving ART will be done.

**Purpose:** The purpose of this research is to determine the prevalence and associated factors overweight and obesity among adults receiving ART in which the result of this study will be distributed to the participants and the concerned stake holders in order to intervene, undesired outcomes. Your involvement is important for the success of this study. You are chosen to participate in the study because you are taking ART in this Hospital. Decision on your involvement will be made by you and only you.You will be asked to answer different questions about you andin addition you will be asked to provider a 6 ml of blood from your vein.

**Risk:** There is a little pain you feel when injecting your vein as usual but there is no psychological risk expected being involved in this study.

**Benefits:** You have the right to know the findings of the study. You will be given advice on the causes and consequences of overweight and obesity and you will also linked to appropriate department for possible treatment and nutritional advice in case you are with undesired result. The study findings will also be used to design and implement control strategies in the study area in the future.

**Incentives:** You will not be provided any incentives to take part in this research.

**Confidentiality:** Your personal information will only be used for the purpose of the study. You will not be personally identified in the study report.

**Participation:** You have to know that your participation is largely based on your willingness and approval. You have the right to say “no” and not participate in the study. You will not be punished if you decide not to participate. If you wish to with draw from this study you can do so at any time without penalty or lose of benefits to which you are otherwise entitled. I would also like to inform you that this study was approved by Wolaita Sodo university research ethical board.

**Confirmation of agreement:** I have read the consent form or the interviewer has read the consent form. I have understood the aim of the study and the things that I have to do if I agreed to participate in the study. I know that my participation is based on my will and have the right not to do so, if I do not want to participate.

Please tell me if you agree or not?

Yes___________ continue the questionnaires page No________ Skip the questionnaires page

Interviewer signature _______________________

Date_____________________________________

**Thank you for your willingness to participate in this study**

## Data collection tool English version

**Questionnaire Code____________________**

**Instructions to the interviewers/data collectors**

This questionnaire contains two parts; Read each part carefully and encircle appropriate number of response. If more than one response is needed, it’s possible to encircle more than one response only in response column.

| **Part I: Socio-demographic factors** | | | | | |
| --- | --- | --- | --- | --- | --- |
| No. | Questions | Response | | Code | Remark |
|  | ID number of the patient | ______________ | |  |  |
| Q101 | Age of the Patients in Year | ______________ Years | |  |  |
| Q102 | Sex of the of the Patients | 1.Male  2.Female | | 1  2 |  |
| Q103 | Marital status | 1.Married  2.Unmarried | | 1  2 |  |
| Q104 | Educational status | 1.No formal education  2.Primary school  3.Secondary school  4.Tertiary school | | 1  2  3  4 |  |
| Q105 | Work | 1.No  2.Yes | | 1  2 |  |
| Q106 | Monthly household income | 1< 1000 ETB  2 ≥ 1000 ETB | | 1  2 |  |
| Q107 | Place of residence | 1.Urban  2.Rural | | 1  2 |  |
| Q108 | Functional status | 1.Working  2.Ambulatory  3.Bedridden | | 1  2  3 |  |
| Q109 | Religion | 1.Orthodox  2.Muslim  3.Protestant  4.Others | | 1  2  3  4 |  |
| Q110 | Ethnicity | 1.Sidama  2.Wolaita  3.Oromo  4.Gurage  5.Others | | 1  2  3  4  5 |  |
| **Part II: Clinical and medication characteristics** | | | | | |
| Q111 | Baseline WHO clinical stage | | 1.WHO stage I  2.WHO stage II  3.WHO stage III  4.WHO stage IV | 1  2  3  4 |  |
| Q112 | CD4 count at ART commencement | | 1.< 200 cells/mm^3^  2. 200-499 cells/mm^3^  3. ≥500 cell/mm^3^ | 1  2  3 |  |
| Q113 | Recent CD4 count | | 1. < 200 cells/mm^3^  2. 200-499 cells/mm^3^  3. ≥500 cell/mm^3^ | 1  2  3 |  |
| Q114 | History of DM | | 0.No  1.Yes  **If Yes**  1.Before ART  2. After ART  **Family History**  1.Yes  0.No | 1  2  1  2  1  2 |  |
| Q115 | Blood pressure | | 1.Systolic BP (mmHg)  2.Diastolic BP (mmHg) | 1  2 |  |
| Q116 | History of HTN | | 0. No  1. Yes  **If Yes**  0.Before ART  1. After ART  **Family History**  0. No  1. Yes | 0  1  0  1  0  1 |  |
| Q117 | Recent Fasting Plasma Glucose (mg/dl) | | ------------------------------- |  |  |
| Q118 | History of TB co-infections | | 0. No  1. Yes | 0  1 |  |
| Q119 | Presence of Opportunistic infection in the past 6 months | | 0. No  1. Yes | 0  1 |  |
| Q120 | Time since HIV diagnosis ( Confirmed date) | | _________________ year |  |  |
| Q121 | Duration of RT experiences | | _________________year |  |  |
| Q122 | Hemoglobin status | | ________________mg/dl |  |  |
| Q123 | Line of ART regimen | | 1. 1^st^ line  2. 2^nd^ line | 1  2 |  |
| Q124 | Exposure to AZT | | 0. No  1. Yes | 0  1 |  |
| Q125 | Exposure to d4T | | 0. No  1. Yes | 0  1 |  |
| Q126 | Exposure to PI | | 0. No  1. Yes | 0  1 |  |
| Q127 | Experiences of NNRTI | | 1.EFV  2.NVP | 1  2 |  |
| Q128 | Drug Adherence status | | 1. Good  2. Fair  3. Poor | 1  2  3 |  |
| Q129 | Baseline BMI status | | 1.< 18.5 kg/m^2^  2.18.5-24.9 kg/m^2^  3.25-29.9 kg/m^2^  4.≥30 kg/m^2^ | 1  2  3  4 |  |
| Q130 | BMI status at the time of the study | | 1.< 18.5 kg/m^2^  2.18.5-24.9 kg/m^2^  3.25-29.9 kg/m^2^  4.≥30 kg/m^2^ | 1  2  3  4 |  |
| Q131 | ART eligibility criteria | | 1.WHO staging  2.Immunologic  3.Both | 1  2  3 |  |
| Q132 | Abdominal obesity ( WHR) | | 0. No  1. Yes | 0  1 |  |
